# Magnitude Representations in Williams Syndrome: Differential Acuity in Time, Space and Number Processing

**DOI:** 10.1371/journal.pone.0072621

**Published:** 2013-08-28

**Authors:** Laurence Rousselle, Guy Dembour, Marie-Pascale Noël

**Affiliations:** 1 Center of Cognitive Neurosciences, Psychological Sciences Research Institute, Catholic University of Louvain, Louvain-la-Neuve, Belgium; 2 Department of Pediatric Cardiology, Saint-Luc University Hospital, Bruxelles, Belgium; Vanderbilt University, United States of America

## Abstract

For some authors, the human sensitivity to numerosities would be grounded in our ability to process non-numerical magnitudes. In the present study, the developmental relationships between non numerical and numerical magnitude processing are examined in people with Williams syndrome (WS), a genetic disorder known to associate visuo-spatial and math learning disabilities. Twenty patients with WS and 40 typically developing children matched on verbal or non-verbal abilities were administered three comparison tasks in which they had to compare numerosities, lengths or durations. Participants with WS showed lower acuity (manifested by a higher Weber fraction) than their verbal matched peers when processing numerical and spatial but not temporal magnitudes, indicating that they do not present a domain-general dysfunction of all magnitude processing. Conversely, they do not differ from non-verbal matched participants in any of the three tasks. Finally, correlational analyses revealed that non-numerical and numerical acuity indexes were both related to the first mathematical acquisitions but not with later arithmetical skills.

## Introduction

The last 30 years, converging lines of evidence suggested that our representation of numerosity is phylogenetically and ontogenetically related to the way we represent other, non-numerical magnitudes. Meck and Church [1] were the first to speculate about a common representation for counting and timing. They observed that both the numerosity and the total duration of a sequence of events could act as an effective stimulus to elicit an appropriate response in rats with cross-stimulus types transfer from numerosities to duration and inversely. This behavioural pattern led them to assume that time and number would be both represented in the same way, as mental magnitudes. Since then, the question of the relationships between numerical and non-numerical magnitudes has generated considerable interest and was extended to the processing of spatial dimensions. In a review published in 2003, Walsh compiled behavioural, neuropsychological and brain imaging results coming from animal and human studies on various types of magnitude processing. He proposed the existence of a central magnitude system for the processing of time, space and numerosity (ATOM, A Theory Of Magnitude ) [2], [3]. This common representation would be shared across species and development and would be located in the parietal neuronal circuitry.

Taking a developmental perspective, several authors defend the idea that non numerical magnitude processing could form the basis on which true numerical concepts develop. This position has its historical roots in the work of Piaget who claimed that numerical concepts are built quite late (around 6–7 years of age) upon children’s ability to process continuous perceptual properties [4]. Of course, it is now widely acknowledged that children show a much more precocious sensitivity to number magnitude than was initially professed by Piaget. But like him, a number of authors now consider that early quantification of discrete and continuous quantities could be initially undifferentiated and represented in terms of overall amount [2], [3], [5], [6]. This hypothesis finds support in studies reporting infants’ and preschoolers’ inability to discriminate or compare numerosities when correlated perceptual variables were rigorously controlled [7]–[11]. A discrete concept of number would develop only later, progressively, possibly through one-to-one correspondence activities which may provide meaningful inputs to apprehend equivalence, ordinality, or even the results of numerical transformations [6]. Walsh (2003) speculated that “*statistical learning of associations between temporal and spatial features of the environment is the means by which this representation* [numerosity] *is learned*” (p.122). Consistent with this scenario, the influence of numerosity over the perceptual quantification of visual arrays was found to increase with age whereas the influence of perceptual cues over numerical quantification was shown to be quite stable or even to decrease during childhood [12]. These divergent developmental trajectories for perceptual and numerical quantification indicated that their automatization is achieved at different paces with perceptual processing reaching maturity earlier than numerical ones.

If spatio-temporal processing forms the basis on which numerical information could gradually be extracted in the course of development, a primitive disability in processing spatial or temporal information should severely compromise subsequent numerical development. This is the prediction made by Simon [13] to explain basic numerical processing impairments in the 22q11.2 deletion syndrome, claiming that a spatiotemporal processing dysfunction “*create a suboptimal foundation for the subsequent development of numerical and mathematical competence, thereby “cascading” impairments into those more academic domains*” (p. 52). Here, this hypothesis was tested in examining numerical and non-numerical magnitude processing in people with Williams syndrome (WS), a neurodevelopmental disorder of genetic origin caused by the microdeletion of 20 to 30 contiguous genes on chromosome 7q11.23. Individuals with WS present quite variable intellectual efficiency ranging from low average to severely impaired (see [14] for a review) with the vast majority of them having mild mental disability [15]–[17]. This genetic syndrome is known for being characterized by a very unequal cognitive profile including a selective damage of spatial cognition and relatively preserved language and facial processing.

Several reasons make thinking that this particular cognitive profile provides a unique opportunity to study the developmental relationships between numerical and non-numerical magnitude processing. First, several studies demonstrated that WS is related to functional and neurostructural abnormalities that are particularly prominent on the dorsal stream, especially in the parietal cortex and the intraparietal sulcus [18]–[22]. These brain regions have been precisely pointed as the locus of overlapping activations during space, time and numerosity processing in brain imaging studies (see [2], [3], [23] for reviews). Interestingly, single-cell recordings in the depth of the intraparietal sulcus of monkeys demonstrated the existence of population of neurons that encodes both numerical and spatial magnitudes [24]. People with WS are thus at risk to present a various magnitude processing deficit due to the structural and functional anomalies of their parietal cortex.

As a matter of fact, their cognitive functioning is indeed characterized by severe impairments in a variety of visuo-spatial abilities supported by the parietal cortex [14], [25]–[34], which contrasted with a far better preserved verbal function [15]–[17], [30], [35], [36]. There is thus a strong probability that people with WS would also experience difficulties in processing spatial magnitudes. Yet, surprisingly, the processing of spatial magnitudes has received very little attention in WS. Visuo-spatial deficits were commonly explored through complex visuo-constructive tasks (i.e. Block Design subtest of the Wechsler scales, puzzle or drawings, or even 3D-geometry) involving a series of different processing steps that still remain poorly specified in the literature (see [27], [37] for reviews). Most of these tasks require good visuo-perceptive and constructive skills, among others, figure ground discrimination, segmentation, perception of orientations, spatial relationships and perspectives, visuo-motor coordination, arrangement of visuo-spatial relationships, planning, monitoring and executive control. Otherwise, studies that focused on visuo-perceptual abilities in WS mainly showed a deficit in the line orientation judgment task [33], [34]. However, to our knowledge, the perceptual processing of one-dimensional spatial dimension (i.e. length or height) has never been systematically examined in WS.

The same is true for the processing of temporal magnitudes as no study selectively focused on time perception in WS. Indirect evidence of preserved temporal representations came from studies demonstrating that people with WS have quite good music perception and rhythmic production abilities taking count of their general cognitive profile [38]–[40]. However, these competences rest on multi-dimensional time and sound processing abilities, including the perception of sounds (i.e. duration, pitch, timbre, and loudness), the frequency (i.e. number of sounds/time unit) or the rhythm (detection of a sequence repetition in a series of sounds).

Finally, a last reason why the cognitive profile in WS is of particular interest to study the relationship between numerical and non-numerical magnitude processing is that people with WS experience particular difficulties with mathematics learning, which is another consequence of their parietal dysfunction [41]–[45]. Their mathematical abilities indeed give the impression to follow a divergent progression trajectory as compared to their reading and spelling skills. Some evidence suggests that mathematical achievement in people with WS stagnate from adolescence at a level that would correspond to the one of a 8 year-old child [46]. Moreover, other clues indicate that mathematical cognition might not develop homogeneously in WS and that some components of mathematical knowledge may be differentially damaged. Regarding arithmetical development for example, some adults with WS become able to verify quite precisely single-digit additions and multiplications (less than 20% errors) with latencies comparable to those of third and fourth grade children, respectively [44]. In production tasks however, they show lower performance in solving timed additive and subtractive arithmetic facts than much younger children matched individually for non-verbal reasoning (Mean chronological age = 6 years; SD = 1 year [45]). A similar example of dissociation was also noted in basic numerical processing development as individuals with WS perform more poorly than controls (matched on non-verbal reasoning) when choosing between two Arabic numbers (one- and two-digits) the one that was closest to a target number. By contrast, they performed better than their matched controls at reading Arabic numbers, suggesting that verbal mathematical skills may be comparatively better developed in WS.

In line with the actual hypothesis formulated to account for the origins of mathematics learning disabilities, this difficulty to process the magnitude of Arabic numbers was thought to result from a primitive failure to represent and process numerosities. Using an habituation paradigm, Van Herwegen et al. [47] indeed showed that 35 month-old children with WS (mean developmental age : 22 months) were unable to discriminate large numerosities differing by a ½ ratio (8 versus 16 dots), an ability widely demonstrated in 6 month-old typically developing babies [48]–[50]. Similar observations have been made in older patients with WS who showed lower sensitivity to numerosity difference when comparing collections of dots (1 to 9 dots) [42]. To date, this is the most primitive deficit that has been identified as the possible source of subsequent numerical cognition disorder in WS.

However, as the processing of one-dimensional spatial and temporal magnitudes was left unexplored so far in individuals with WS, it not possible to know whether their reduced sensitivity to numerical magnitudes is specific to the numerical domain or originates from a primitive disability to process non numerical magnitudes, especially spatial ones. In the present study, this issue was addressed in examining spatial, temporal and non-symbolic numerical processing in individuals with WS and two groups of typically developing (TD) children matched either on verbal or on non-verbal mental age. These processes were assessed through magnitude comparison tasks that focused on length, duration and numerosity respectively. Among the variety of spatial and temporal dimensions, length and duration were selected as their interaction with number magnitude processing is well documented in the literature [51]–[60]. In the spatial task, participants were instructed to compare the length of two lines while in the temporal task, they were asked to compare the duration of two sounds. To equate as much as possible the memory load in the two non-numerical magnitude comparison tasks, the two lines were presented sequentially (as were the sounds in the auditory modality). In an attempt to replicate previous results showing lower sensitivity to non-symbolic numerical difference in WS, numerosity processing was examined using a classical non-symbolic task in which participants had to compare the numerosity of two visual arrays. In the three tasks, the magnitude ratio (i.e. the ratio between the line lengths, the sound durations or the numerosities) was manipulated so that participants were presented with stimulus pairs engaged in less and less discriminable ratios along the experiment (from ½ to 8/9). Varying the magnitude ratios between the stimuli to be compared allowed us to calculate the Weber fraction, an index of the perceptual/numerical acuity in each task. The Weber fraction can be defined as the smallest change in magnitude that can be reliably discriminated. As all tasks recruit, to some extent, verbal and visuo-spatial working memory resources, participants were administered additional measures taxing the two storage components of short-term memory, namely, the phonological loop and the visuo-spatial sketchpad (VSSP) [61], [62].

Finally, mathematical achievement was examined using relevant measures selected to catch the wide range of mathematical abilities of our sample, from the acquisition of counting and cardinality to more complex arithmetic skills. The comparison with verbal and non-verbal matched TD children should allow determining whether mathematical development in people with WS corresponds to what would be expected on the basis of their verbal and nonverbal cognitive profile. Furthermore, in the present framework, assessing mathematical achievement offers the opportunity to examine how far it is related to numerical and non-numerical acuity. Numerical acuity has already been found to predict later mathematical achievement [63]–[65]. Recently, the precision of both numerical and spatial (cumulative area) magnitude representations was shown to correlate significantly with advanced mathematical competences [66]. Likewise, if mathematical competences actually build on non-numerical magnitude processing, we should find a relationship between spatio-temporal acuity and mathematical achievement.

To sum up, the aim of the present work is to examine the specificity of the numerical magnitude processing deficit in WS. If participants with WS have difficulty to process all kinds of magnitude, they should exhibit poorer acuity (i.e. higher Weber fraction) in processing numerical, spatial and temporal dimensions. This pattern of results would support the view of a developmental continuity between numerical and non-numerical magnitude processing deficit [2], [3], [13]. Conversely, if their representation of non-numerical magnitudes is preserved, they should have no difficulty to process continuous spatial or temporal information.

## Method

### Ethics Statement

The experiment was conducted in accordance with the Declaration of Helsinki and the experimental protocol was approved by the regional ethical committee for biomedical research of the Department of Medicine of the Catholic University of Louvain which is in charge of the investigation in patients (Record number : B403201111579). As none of our participants were legally competent, their parents were asked to give their written informed consent allowing their child to participate in the study. Participants themselves were asked orally by their parents if they would accept to participate and all of them agreed.

### Participants

Twenty children and adults with WS participated in this study. They were recruited through the department of pediatric cardiology of the Saint-Luc University Hospital in Brussels, Belgium and through the French-speaking Williams Syndrome Foundation of Belgium. The clinical diagnosis of WS was confirmed for all patients by the fluorescence in situ hybridization test (FISH) or by the Multiplex Ligation-dependent Probe Amplification method (MLPA). The mean chronological age for participants with WS was 22 years 1 month (range : 5;6 years to 52;10 years, 10 females).

Forty typically developing (TD) children were then recruited and individually matched to participants with WS. As most individuals with WS present mild intellectual disability (ranging from low average to severely impaired intellectual efficiency), it was not possible to test them with the scale corresponding to their chronological age and consequently, not possible to compute standard scores either. Accordingly, half of TD children were matched on verbal developmental level (TDv children) using the mean raw scores of the Similarity and the Vocabulary subtests of the Wechsler Intelligence Scales for children [67], [68]. The other half was matched on nonverbal mental development (TDnv children) using the raw scores of the Block Design and the Picture Concept subtests of the Wechsler Intelligence Scales for children. Matching participants using raw scores equals matching them on the basis of their developmental age. The mean chronological age was 7 years 6 months (Age range: 4;6–11;8 years; 17 females) for TDv children and 6 years 1 months (Age range: 3;8–10;4 years; 15 females) for TDnv participants.

### Tasks and Stimuli

#### IQ measures

All participants were administered four subtests of the Wechsler intelligence scales for children: (1) the Similarity subtest, a verbal conceptual matching task in which the common conceptual features relating two words has to be found, (2) the Vocabulary subtest, a verbal definition task, (3) the Picture Concept subtest, a picture-based conceptual matching task with no visuo-spatial content and finally, (4) the Block Design subtest which requires reproducing visuo-spatial patterns with blocks. Depending on the abilities of participants with WS, the administered subtests were drawn either from the Wechsler Preschool and Primary Scale of Intelligence-3^rd^ edition [68] or from the Wechsler Intelligence Scale for Children-4^th^ edition [67]. The same subtests were then administered to each of their matched peers. Verbal and nonverbal developmental levels were respectively estimated as the mean of the raw scores in the Similarity and Vocabulary subtests and in the Picture Concept and Block Design subtests.

#### Working memory

The phonological loop and the VSSP were individually examined in tasks that did not require the recall or manipulation of numerical contents. The phonological loop capacity was assessed in a forward letter span task. Participants were instructed to listen to a sequence of letters and to repeat them immediately in the same order. Letters were read at the rate of one per second. No repetition was allowed. Sequences consisted of monosyllabic consonants with no repetition within any sequence. The first sequences included two letters and were then followed by sequences of increasing length (3 to 9 letters). For each sequence length, there was a maximum of three trials, out of which only the two best trials were scored. Participants who succeeded at repeating two sequences of *n* letters were given sequences of *n +1* letters at the next trial. The task was stopped when a participant failed at two out of the three trials for a given sequence length. Each correct response was credited with one point.

The VSSP was assessed with a *two-dimensional* visuo-spatial span task inspired from the Corsi block test. Participants were presented with a blank matrix and were instructed to remember the location of a series of cells touched one by one, by the examiner. They had to indicate which cells were touched by placing tokens in the right location in the matrix (order was of no importance). Note that the path drawn by the examiner in the matrix did not follow any regular pattern and included a minimum of number of crossings (maximum 1). For each trial, participants were given the exact number of tokens to be placed. Trials were of increasing complexity as the size of the matrix and the number of touched cells increased during the task. The lower level of difficulty corresponded to a 2×2 matrix with two touched cells and the higher level included a 4×5 matrix with 10 touched cells. There were different entry points in the protocol depending on the age (i.e. 3–4 year-olds started with 2 tokens to be placed in a 2×3 matrix; 5–6 year-olds with 3 tokens to be placed in a 3×3 matrix and older participants with 4 tokens to be placed in a 3×4 matrix). For participants with WS, the entry point was estimated from their non-verbal developmental level. If a participant did not answer accurately to the first two items of his age level, he was administered the items from the lower age group until he succeeded the two first items and the more complex items were then represented a second time in the order. The maximum score was then granted for lower levels of difficulty. As for the letter span task, each level of difficulty included a maximum of three trials, out of which only the two best were scored. Participants had to succeed in two trials of the same difficulty level to access to a higher difficulty level (larger matrix and/or larger number of touched cells). The task was stopped when a participant failed at two out of the three trials for a given difficulty level. Each correct response was credited with one point.

### Mathematical Development

Given the heterogeneity of mathematical abilities in our sample, three kinds of tasks were administered to assess the different levels of mathematical development: the “give a number” task, the pictorial additive fluency and single-digit arithmetic fluencies (without picture).

The “give a number” task was administered to assess the understanding of the cardinal value of number words and the ability to use the counting procedure to give large numbers. The procedure was modeled on the task developed by Le Corre and Carey [69]. Small colored stones were placed on the table. Participants were first asked to give *one* stone in the hand of the examiner. For the six first number words, participants who succeeded at giving *n* number of stones were asked to give *n* +1 on the next trial but those who failed were requested to give *n* –1 on the subsequent trial. From the number word *six,* participants could be asked for 8, 10 and finally 14 stones if they succeed at giving the requested number. A maximum of three trials were administered by number word. The task continued up to the first number that the child failed to give correctly at least two out of three times. As in Wynn’s procedure [70], [71], participants were allowed to make a single counting error, that is, they could be credited even when they had actually given *n* ±1, provided that they used counting to produce the set. The cardinal developmental level was determined as the highest number that they could correctly give at least twice.

The pictorial additive fluency task was adapted from the one developed by Noël [72] to test the first arithmetic skills in preschoolers. A series of ten additions with pictorial support was proposed. Each problem was presented orally and was accompanied with a drawing illustrating the numerosity of the first operand (e.g., “Look, here are two fishes; if two more come, how many fishes will there be?”). The set included five ties (1+1, 2+2, 3+3, 4+4, 5+5) and five additions with the larger addend presented first (2+1, 3+2, 4+3, 5+4, 6+5). Items were of increasing complexity with smaller sums presented first and larger sums presented last (sum order: 2, 4, 3, 6, 5, 7, 8, 10, 9 and 11). Participants had 150 seconds to solve as many problems as possible. Instructions emphasized both speed and accuracy. They were told that they can use tokens if it helps. The timer started at the end of the reading of the first problem. The second part of the problem that included the second addend (not illustrated) could be repeated if necessary. No feedback was given during the task except for the first item (1+1) in case of a wrong answer. The next item was presented every time a participant gave an answer or seemed to be blocked more than 20 seconds on one item. Each correct response was credited with one point. If a participant correctly solved the 10 items before the time limit, 1 bonus point was given for each interval of 5 seconds saved on the allotted time.

Finally, the single-digit arithmetic fluencies consisted of three tasks involving simple additions, subtractions and multiplications. For each operation, participants were presented with a sheet of written arithmetic problems and had 150 seconds to solve as many problems as possible (written response). Additions and multiplications problems were drawn from all possible combinations of the integers 1–9 and the set of subtractions was the exact counterpart of the addition set. These combinations resulted in a total set of 81 problems for each operation, respectively. In each task, the experimenter scored the number of correct responses given in the allotted time and the number of errors.

### Magnitude Comparison Tasks

Numerical and non-numerical magnitude comparison tasks were carried out on a tablet PC (HP Elitebook 2740p, Screen: 12.1-inch WXGA (1280×800)). Stimuli were presented on a blue navy background with the E-Prime experimental software (Version 1.1, Psychology Software Tools, Inc., Pittsburgh, PA). The three tasks required participants choosing between two possible responses, one presented on the left and the other on the right side of the screen. The tactile screen surface was divided by an invisible vertical midline defining two equal response zones. To give their answer, participants were instructed to touch the screen with the tactile pen on the side of the correct response. Instructions emphasized both speed and accuracy. They were repeated as often as necessary to keep participants on task.

**Figure 1:**
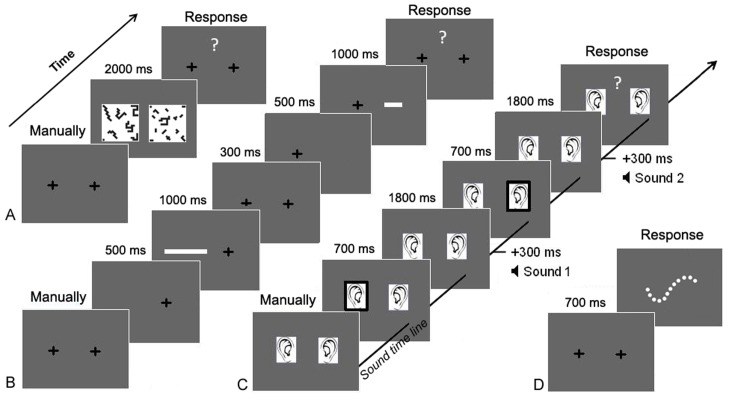
Timing of events during a trial. From left to right, each panel shows the succession of events during a trial in the numerosity (A), the length (B), the duration (C) comparison tasks and the speeded counting task (D).

In the *numerical comparison task*, participants were presented with two white boxes containing black pieces of puzzle and were asked to compare the numerosities of the two collections. To prevent participants from basing their judgement on perceptual non numerical dimensions, the numerosity and the cumulated black area were manipulated in two congruity conditions. In congruent trials, the larger array in number had also the larger cumulated black area while in incongruent trials, the larger collection in number had the smaller cumulated black area. The form of the individual pieces was manipulated so that the variations of cumulated black area were completely confounded with those of cumulated individual perimeter (i.e. sum of individual piece perimeters) and brightness. To avoid the larger collection in number being systematically the one with the smaller elements, the area of the smaller and larger pieces was the same in both arrays to be compared. Finally, the external perimeter of collections (formed by the most external pieces) was equated for all trials. The trial started with the presentation of two fixation crosses respectively displayed on the left and right side of the screen. When the participant was judged to be visually attending to the display, the two collections were simultaneously presented on the screen for two seconds, one on the left and the other on the right side of the screen, covering both a visual angle of approximately 24.8° ×9.1° (see Figure 1A). Participants had to touch the screen on the side of the box that contained more pieces. Instructions emphasized that the size of the pieces were of no importance. Participants were encouraged to find the box that enclosed more different pieces and were instructed not to count the pieces as they would not have the time to. They could respond as soon as they got the answer with no time limit after stimuli disappearance. If no response was given within about 3 seconds, participants were prompted to do so by asking “so, which box contained more pieces?”.

In the *length comparison task*, participants had to compare the length of two white lines presented successively. The trial started with the presentation of two red fixation crosses respectively displayed on the left and right side of the screen. The line size varied between 2.3° and 10.3° of visual angle. When the participant was judged to be visually attending to the display, the experimenter triggered the disappearance of the left cross followed by the appearance of the first line, on the left, for 1000 ms. Then, the left fixation cross reappeared and the right cross disappeared and was replaced by the second line on the right side of the screen for 1000 ms (see Figure 1B). Participants had to touch the screen on the side of the longest line. They could respond as soon as the second line was displayed with no time limit after its disappearance. If no response was given within about 3 seconds, participants were prompted to do so by asking “so, which line was the longest?”.

Finally, in the *duration comparison task*, participants had to compare the duration of two identical sounds presented in succession (Range = [225–1350 ms]; audio format: 44100 Hz, 32 bits, Mono). To attribute a location to the played sounds, two ears were respectively displayed on the left and right side of the screen throughout the task (i.e. black and white drawings of ears covering both 18.7° of visual angle, see Figure 1C). The trial was initiated by the experimenter when the participant was judged to be visually attending to the display. The left ear was first surrounded by a red frame for 700 ms. Three hundred milliseconds after the disappearance of the left red frame, the first sound was played bilaterally by the computer speakers. After a variable delay (between 150 and 1125 ms), a red frame was displayed for 700 ms around the right ear. The second sound was then played 300 ms later. Participants had to touch the screen on the side of the ear that “heard” the longest sound. They could give a response from the beginning of the second sound with no time limit. If no response was given within about 3 seconds, participants were prompted to do so by asking “so, which ear has heard the longest sound?”.

In the three tasks, the quantitative ratio between the magnitudes to be compared was of increasing complexity along the task. Participants always started with stimulus pairs varying according to the two easiest ratios, that is, ½ and 2/3. Less and less discriminable ratios were then progressively introduced (3/4, 5/6, 7/8, and finally 8/9), depending on participant’s correct response rate for each ratio. Pairs of consecutive ratios were always intermixed with each other so that stimulus pairs of one ratio were never presented alone. The task was discontinued when a participant performed at chance level for two on three consecutive ratios. Thus, the 6 ratios were not administered to all participants. This procedure was adopted to take into account participant’s individual limits regarding their sensitivity to magnitude differences but also their own attentional capacities. Indeed, presenting participants too many ratios that they are not able to discriminate could be discouraging. This could lead them to adopt “guessing” strategies [73] that would have brought a lot a noise in the data, including on easy ratios that could be in fact well discriminated.

As reported in Table 1, two different pairs of magnitudes were used for each ratio. The side of the correct response was counterbalanced: each pair appeared four times, twice with the larger magnitude on the right side and twice with the larger magnitude on the left side. When all ratios were presented, participants were administered a total of 48 stimulus pairs in each task (2 pairs ×2 sides ×2 presentations×6 ratios). Along the experiment, pairs were presented in a pseudo-random order (i.e. no identical pairs in two consecutive trials, no more than three consecutive correct responses on the same side and no more than two identical ratios in succession).

**Table 1:** Pairs of Magnitudes Presented in the Numerical and Non-Numerical Comparison Tasks.

	Ratios					
	1/2	2/3	3/4	5/6	7/8	8/9
Numerosities	7–14	6–9	6–8	5–6	7–8	8–9
	8–16	10–15	12–16	10–12	14–16	16–18
Lengths^a^	70–140	60–90	60–80	50–60	70–80	80–90
	80–160	100–150	120–160	100–120	140–160	160–180
Durations^b^	525–1050	450–675	450–600	375–450	525–600	600–675
	600–1200	750–1125	900–1200	750–900	1050–1200	1200–1350

Note. ^a^Lengths are expressed in millimetres.

b Durations are expressed in milliseconds.

Before beginning each task, participants performed six practice trials with pairs of magnitudes differing by a 1/3 ratio to check the understanding of the instructions. Test trials were presented only if the participant performed accurately on at least five practice trials. Practice trials could be administered up to three times if a participant did not reach the criterion of 5/6 correct response.

### Processing Speed Assessment

Two additional processing speed tasks were administered as control measures. First, a *stimulus detection task* was used as a measurement of general processing speed to inquire whether processing speed differences might account for participants’ performance in the magnitude comparison tasks. A white dot appeared on the left or right side of the screen and participants were asked to touch the dot with the tactile pen as fast as they could. This task provide a reliable measure of the time necessary for stimulus detection and response production. Second, a *speeded counting task* was designed to measure participants’ counting speed on the computer screen. The aim was to determine whether participants would be able to count fast enough to enumerate precisely the number of pieces presented in the numerical comparison task. They were presented with eight string of 6, 7, 8, 9, 12, 14, 16 and 18 dots in a fixed pseudo random order (see Figure 1D) and were asked to count them as fast as they can and to tell how many dots were displayed. They were specifically asked to count dots one by one and were told that they could use their fingers if they wanted to. Dots were arranged successively to facilitate the distinction between counted and to be counted items. The counting speed was determined for each correct trial as the time taken to count *one* item (i.e. total counting time divided by the number of dots to be counted for each correct trial). The average counting speed by item was then calculated over all correct trials. In the WS group, one participant was unable to give a single correct answer.

## Experimental procedure

Participants were tested individually in a quiet room. Testing was completed in two 75 minute sessions, approximately, depending on participant’s performance and attentional level. Participants could take a break any time they needed or when the experimenter felt it was necessary. The first session started with the four IQ subtests (the Block Design, the Similarity, the Picture Concepts and then, the Vocabulary subtest) followed by the two working memory subtests, that is, the visuo-spatial span, and then the letter span task. The tasks assessing mathematical development opened the second session in the following order : the “give a number” task, the pictorial additive fluency and then, the single-digit arithmetic fluencies (i.e. addition, subtraction and then, multiplication). Finally, the order of the four quantification tasks (i.e. the speeded counting and the three magnitude comparison tasks) was balanced following a latin square design. The stimulus detection task was administered at the end of the second session. Other tasks were run during the first and the second session but they were part of another study.

## Results

### Descriptive Measures

Table 2 reports mean chronological age, mean working memory scores and mean counting speed by item in the control task (in milliseconds) for each group. Table 3 reports participants’ mean performance in the tasks assessing mathematical development. Given the heterogeneity of math achievement levels in our samples, the mathematical development tasks could not be administered successfully to all participants. One participant with WS was not administered the Pictorial additive fluency task as he was unable to calculate and failed to give any answers. Likewise, ten participants with WS were not administered any of the single-digit arithmetic fluencies as they were unable to perform even a single calculation presented in this symbolic format whatever the operation. As a result, their verbal and nonverbal-matched controls were not administered these tasks either. In addition, six TDnv participants were too young to perform the single-digit arithmetic fluencies. The number of participants thus varied depending on the tasks and the groups included in the analysis, as displayed in Table 3. Paired-samples T-tests were run to compare each WS participant to his verbal and nonverbal-matched TD peer. Unless otherwise noted, the pattern of group differences across task was the same using paired-sample Wilcoxon non parametric statistics. Predictably, participants with WS were significantly much older in chronological age than both the TDv and TDnv children they were matched to, *t*s(19) >5.64, *p*s <.001.

**Table 2:** Mean Chronological Age and Mean Performance in Working Memory, Processing Speed and Counting Speed by Group.

			WS		TDv		TDnv	
		N	Mean	SD	Mean	SD	Mean	SD
Age (months)		20	265.4	139.4	90.4**	22.2	72.8**	21.5
Working Memory	Visuo-spatial span	20	8.1	3.3	11.6**	2.9	9.4	3.3
	Letter span	20	5.2	1.8	5.9	1.0	5.2	1.4
Processing speed (ms)		20	821	235	753	177	886	250
Counting speed (ms)		19	1081.9	460.5	641.8**	164.4	996.6	430.7

Note. **p<.001.

**Table 3:** Mean Performance in the Mathematical Tasks by Group.

			WS		TDv			WS		TDnv	
		N	Mean	SD	Mean	SD	N	Mean	SD	Mean	SD
Give a number task		20	11.6	4.3	13.8*	0.9				12.4	3.2
Pictorial additive fluencies	CR	19	6.8	3.3	9.5**	1.2	15	6.3	3.4	7.6	2.6
	Errors	19	2.6	3.1	0.2**	0.5	15	3.1	3.3	1.5(*)	2.0
	Bonus	19	3.3	5.8	8.7**	5.3	15	2.4	5.1	4.5	5.9
	Total^a^	19	10.1	8.2	18.2**	6.3	15	8.7	7.5	12.1	7.8
Additive fluencies	CR	10	9.0	5.4	19.0*	9.2	4	10.8	1.9	21.5*	5.4
	Errors	10	1.4	1.4	0.5	0.7	4	2.3	1.7	0.3	0.5
Subtractive fluencies	CR	10	6.0	4.2	16.5**	7.0	4	8.5	3.7	18.0*	3.6
	Errors	10	1.3	1.3	0.6	0.7	4	1.3	1.5	.25	0.5
Multiplicative fluencies	CR	10	4.3	3.2	13.6*	9.5	4	5.3	1.3	13.5	8.9
	Errors	10	1.3	1.5	0.9	0.7	4	2.3	1.7	1.8	1.7

Note. CR = Correct responses.

** p<.001,

* p<.05, (*) p = .06. N = Number of pairs of participants included in the analysis.

a Total correct with bonus credit.

### Comparison with TDv Children

Participants with WS did not differ from their verbal controls on estimated verbal developmental level, *t*(19) = −.22, *p*>.10, nor on the two verbal IQ subtests, Similarities : *t*(19) = 1.13; Vocabulary : *t*(19) = −1.35, both *p*s >.10, confirming that both groups had equivalent verbal developmental level. In non-verbal IQ subtests, participants with WS exhibited significantly lower performance in the Block Design, *t*(19) = −5.81, *p*<.001, *η* ^2^ = .64, as well as in the Picture Concept subtests: *t*(19) = −2.27, *p*<.05, *η* ^2^ = .21. This resulted in a lower mean nonverbal developmental level in the WS group, *t*(19) = −5.79, *p*<.001, *η* ^2^ = .64. In working memory, participants with WS performed significantly lower than the TDv group in the visuo−spatial span task, *t*(19) = −5.75, *p*<.001, *η* ^2^ = .65, but the difference between the two groups failed to reach significance in the letter span task, *t*(19) = −1.82, *p* = .09.

As expected, participants with WS performed lower in all mathematical development tasks compared to TDv children. They made more errors in giving a requested number of objects, *t*(19) = −2.5, *p*<.05, *η* ^2^ = .25, or in solving pictorial additions, *t*(18) = 3.80, *p*<.001, *η* ^2^ = .45. Moreover, in this last task, they calculated much slower as they gave fewer correct responses in the allotted time, *t*(18) = −3.85, *p*<.001, *η* ^2^ = .45, and received lower bonus credit, *t*(18) = −4.5, *p*<.001, *η* ^2^ = .53. Qualitatively, 15/19 TDv participants correctly solved the 10 problems before the time limit in this task while 14/19 participants with WS did not, *χ* ^2^(1) = 10.6, *p* = .001. Similarly, in the single-digit arithmetic fluencies, they were significantly slower in solving additions, subtractions and multiplications, *t*s(9)<−3.09, *p*s <.05, *η* ^2^ = .51, .78, .52, respectively. They also tended to make more errors in additions and subtractions but none of the error group differences in the single-digit arithmetic fluencies reached statistical significance, *p*s <.10.

Finally, the two groups exhibited comparable general processing speed, *t*(19) = 1.18, *p*>.10, but participants with WS counted much slower than their verbal-matched peers in the speeded counting task, *t*(18) = 4.66, *p*<.001, *η* ^2^ = .55. Most importantly, it should be noted that the counting speed was very slow in both groups despite the sequential arrangement of the dots to be counted (Range = [602–2105 ms] and [437–1048 ms] in the WS and TDv groups, respectively). As the arrays to be compared in the non-symbolic numerical task included at least 11 pieces, participants would not have been able to count the pieces in 2 seconds with such a slow counting speed.

### Comparison with TDnv Children

Unsurprisingly, participants with WS had significantly higher estimated verbal development than TDnv controls, *t*(18) = 2.31, *p*<.05, *η* ^2^ = .23, even if the group differences reached significance only in the Vocabulary subtest, Vocabulary : *t*(18) = 2.06, *p* = .05, *η* ^2^ = .19; Similarities: *t*(19) = 1.72, *p* = .10 (the group effect in the Vocabulary subtest was marginal using the Wilcoxon signed rank test, *Z* = −1.7, *p* = .09). Importantly, the two groups exhibited comparable nonverbal developmental level, *t*(19) = .30, *p*>.10, and did not differ from each other on any of two nonverbal IQ subtests, Block Design, *t*(19) = −1.17; Picture Concept: *t*(19) = 1.15, *p*s >.10. There was no difference between groups in any of the working memory tasks, −1.68< *t*s(19) <.09, *p*s >.10.

Regarding mathematical development, the two groups had comparable performance in the give-a-number task, *t*(19) = −1.35, *p*>.10, but there was a marginal significant difference between groups in the pictorial additive fluency task. Both groups correctly solved a similar number of pictorial additions, *t*(14) = −1.43, *p*>.10, and received comparable bonus credit, *t*(14) = −1.33, *p*>.10, but participants with WS tended to commit more errors, *t*(14) = 2.01, *p* = .06, *η* ^2^ = .22. Respectively, 12/15 participants with WS and 8/15 TDnv participants failed complete the task within the time limit in this task, a difference that is not significant *χ* ^2^(1) = 2.4, *p*>.10. Due to their young age, only a small number of TDnv participants were able to complete the other arithmetic fluency tasks. In spite of this, people with WS were found to be significantly slower in solving single-digit additions and subtractions, *t*s(3)<−3.67, *p*s <.05, *η* ^2^ = .82 and.90, respectively (these group differences were marginal with the Wilcoxon signed rank test, *Z* = 1.83 and 1.84, respectively, *p* = .07). They also tended to make more errors in additions and subtractions but this group difference was not large enough to reach significance given the small number of subjects included in this analysis, *t*s(3) = 2.19 and 1.73, respectively, *p*s >.10. There was no group difference in the multiplicative fluency task, in general processing speed or in the speeded counting task, *p*s >.10.

### Magnitude Comparison Tasks

#### Weber fraction

**Figure 2:**
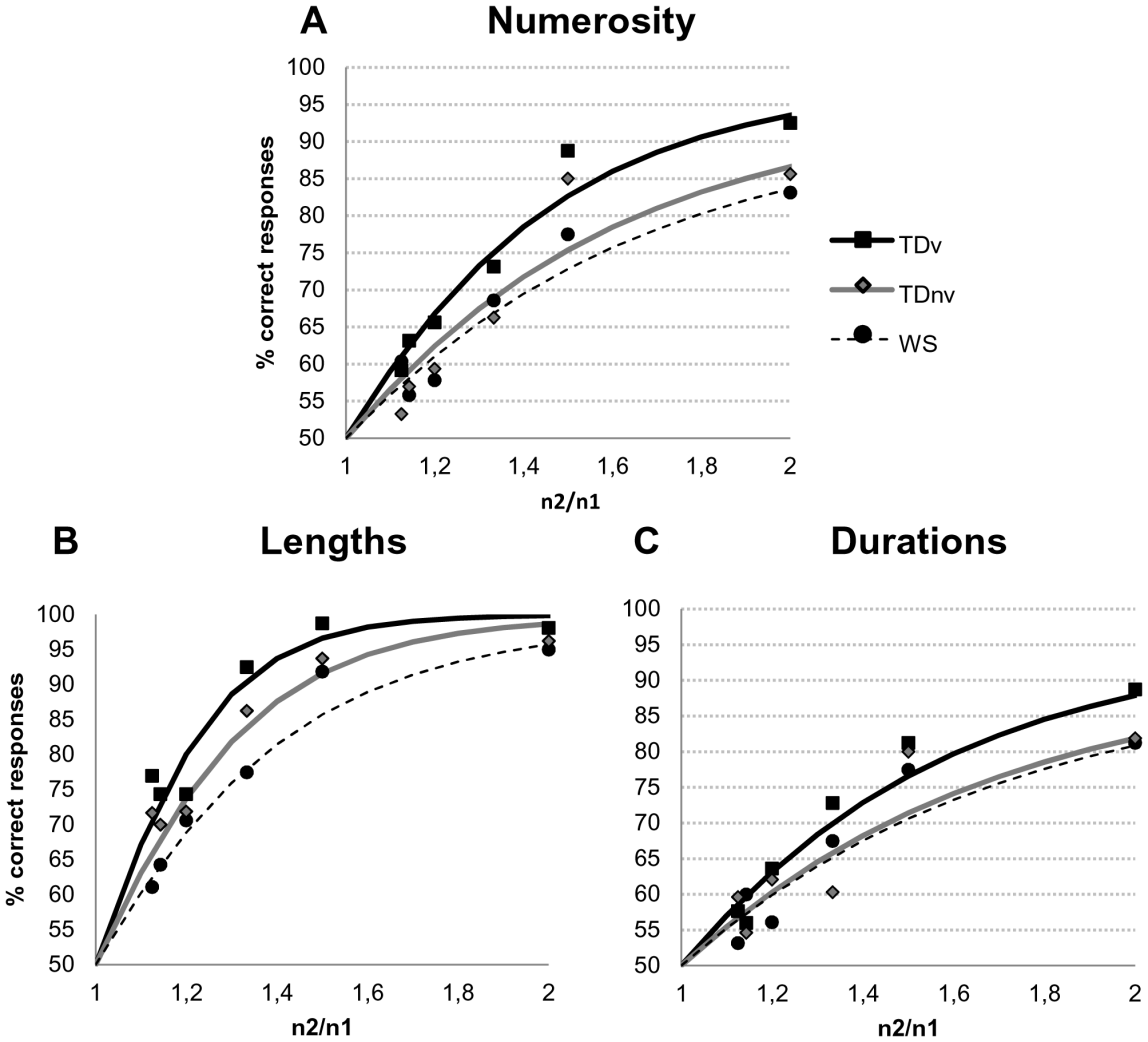
Accuracy data as a function of the ratio. Each panel respectively shows the percentage of correct responses as a function of the ratio in the numerical (A), spatial (B) and temporal (C) comparison tasks presented with logistic regression curves.

As displayed in Figure 2, participants’ performance varied as a function of the ratio between the magnitudes to be compared. In order to assess the precision of the underlying magnitude representations, the Weber fraction (*w*) was estimated individually from the participants’ correct responses in each task. There are several ways to define a Weber fraction and the approach taken here is the one inspired by Pica et al. [74] and Halberda and Feigenson [73] (see Supplement S1 for an extensive description of the Weber fraction estimation method). As our group samples were extremely disparate in terms of chronological and developmental age, it was important to compare each WS participant to his own verbal matched control. Such paired comparison was not possible using an analysis of variance model. Therefore, Weber fraction were analysed separately in each task using paired-sample T-tests.

**Figure 3:**
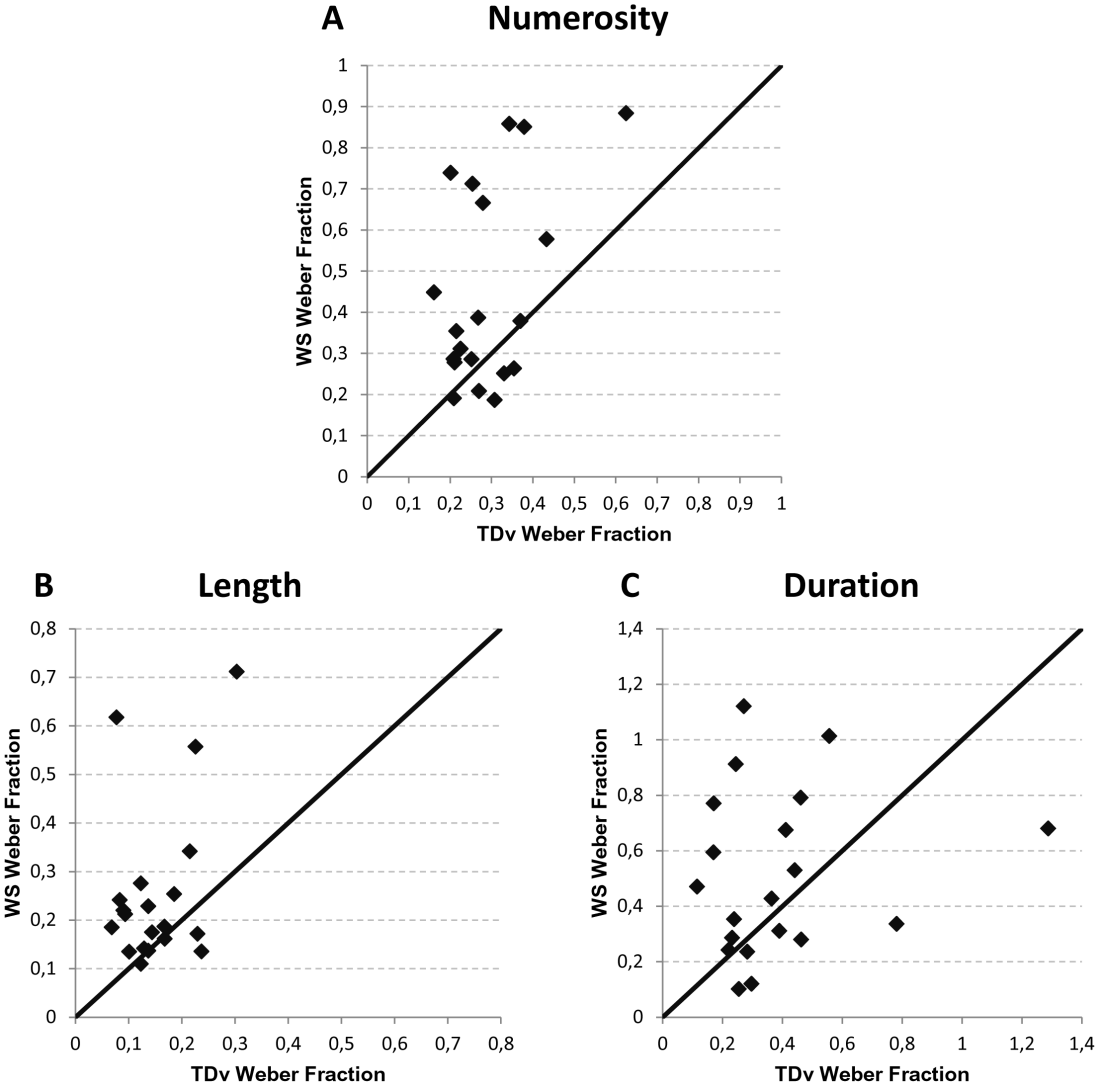
Within-pair Weber fraction associations. Each panel shows the correspondence between Weber fractions within each WS-TDv pair of participants in the numerical (A), spatial (B) and temporal (C) comparison tasks, respectively.

Mean Weber fractions by task and by group are displayed in Table 4. Compared to TDv children, paired-sample T-tests showed significant group effects in the length, *t*(19) = 3.03, *p*<.01, *η* ^2^ = .33, and in the numerical comparison tasks, *t*(19) = 3.38, *p*<.005, *η* ^2^ = .38. In both tasks, the Weber fractions were higher for participants with WS which means that they exhibited a lower sensitivity to numerical and spatial magnitude differences than TDv children. However, these two groups did not differ while comparing duration, *t*(19) = 1.39, *p*>.10. These results were further confirmed using non-parametric statistics. Paired-sample Wilcoxon signed rank test showed a significant group effect in the numerical, *Z* = −2.7, *p*<.01, and the spatial comparison, *Z* = −2.96, *p*<.005, but not in the temporal comparison task, *Z* = −1.42, *p*>.10. Figure 3 illustrates the distribution of the Weber fractions within each WS-TDv pair of participants. The central axis represents the midline on which the point would be located if both the participant with WS and his matched peer would have obtained equal *w*. The space above the axis includes pairs for which the *w* was higher for the participant with WS while the space under the axis includes pairs for which the *w* was higher for the TDv participant. In the numerical and the spatial comparison tasks, most participants with WS obtained higher *w,* and were thus less able to discriminate numerical and spatial magnitude variations than their verbal matched TDv peers. The Weber fraction discrepancy in favour of the TDv children was thus very representative of the group differences and not solely the fact of a subgroup of verbal-matched pairs of individuals. In contrast, the WS-TDv pairs are more evenly distributed around the central axis in the duration comparison task, illustrating that some participants with WS had higher while others had lower sensitivity to temporal difference compared to their matched TDv control, with no significant difference between groups.

**Table 4:** Weber Fraction by Task and by Group.

	WS		TDv		TDnv	
	Mean	SD	Mean	SD	Mean	SD
Numerosity	0.46	0.24	0.29**	0.10	0.40	0.15
Length	0.26	0.17	0.15**	0.06	0.20	0.08
Duration	0.51	0.30	0.38	0.26	0.49	0.28

Note. **p<.01,

* p<.05.

Paired-sample T-tests did not reveal any difference between participants with WS and TDnv children in the three tasks, .26< *t*s(19) <1.45, *p*s >.10, indicating that they had comparable sensitivity to numerical and non-numerical magnitude differences.

As participants with WS exhibited lower performance in the visuo-spatial span task compared to TDv children, the significant group effect in the numerical and the spatial comparison task could be related to within-pair differences in participants’ VSSP capacities. The VSSP is supposed to be recruited in both the numerical and the spatial tasks which require holding on in the visuo-spatial memory the number or the length of the stimuli to be compared. Therefore, the within-pair differences were computed in the numerical and the spatial comparison tasks (Weber fractions) as well as in the visuo-spatial span task. Pearson correlations were then calculated between these differences. There was no correlation between the visuo-spatial span differences and the Weber fraction differences in the numerical (respectively, *r = *.11, *df* = 38, *p*>.10), or the length comparison task (respectively, *r* = −.04, *df* = 38, *p*>.10). Consequently, the group effects reported in the numerical and the spatial comparison task could not be attributed to differences in the VSSP capacities.

### Link with Mathematical Development

A related concern in this study was to examine the relationship between numerical and non-numerical acuity and mathematical achievement. Multiple tasks had to be used to catch the wide range of mathematical achievement level in our samples. As many of them constitute overlapping measures of mathematical skills, principal components analyses were carried out to reduce the number of variables to a smaller set of composite variables representative of participants’ mathematical achievement. Based on the number of individuals who completed each mathematical development task (see Table 3), two separate principal component analyses were conducted, each of which converged fittingly toward a single component solution. The first component extracted could be labelled the *precocious math index* and accounted for 87.5% of the variance in the give-a-number task and the pictorial additive fluencies (number of correct responses). The second component, labelled the *arithmetic fluency index*, explained 90.8% of de variance in the three single-digit arithmetic fluencies (addition, subtraction and multiplication).

The relationships between numerical and non-numerical acuity and the two extracted components of mathematical achievement were then appraised through Pearson correlational analyses. The three quantitative acuity indexes correlated negatively with the the precocious math index, *r*(53) = −.42, −.71 and −.52, for the spatial, temporal and numerical *w* respectively, *p*s <.001. Moreover, the arithmetic fluency index correlated significantly with spatial acuity, (*r*(24) = −.41, *p*<.05, but not with the two other acuity indexes, *ps* >.05. To ensure that these correlations were not mediated by general cognitive abilities, partial correlation controlling for verbal and non-verbal developmental age differences were conducted. For each subtest of the Wechsler intelligence scales, developmental age was estimated as the higher age for which a given raw score correspond to a standard score of 10. Verbal developmental age was estimated as the mean of the developmental ages obtained for the Similarity and Vocabulary subtests while non-verbal developmental age was estimated as the mean of the developmental ages obtained for the Cube Design and Picture Concept subtests. Correlations between the three acuity indexes and the precocious math index resisted the introduction of the covariates, *r*(49) = −.28, −.59 and −.44 for the spatial, temporal and numerical *w*, respectively, *p*s <.05. However, none of the acuity indexes correlated with the arithmetic fluency index anymore, *p*s >.05.

## Discussion

The present work addressed the question of a developmental link between numerical and non-numerical magnitude processing. This issue was examined in people with WS, a genetic syndrome known to associate particular difficulties with math learning and visuo-spatial processing. As a deficit of numerical magnitude processing deficit was already reported in people with WS [42], [47], the aim of this study was to determine whether this magnitude processing deficit is specific to the numerical domain or extends to other, non-numerical magnitudes such as space and time.

People with WS exhibited significantly higher Weber fraction than their verbal matched peers in both the numerosity and the length comparison tasks while no such difference appeared in comparing durations. These differences in numerical and spatial acuity were not solely the fact of a few couples of participants as they are observed in most WS-TDv pairs (contrary to the Weber fraction differences in the duration comparison task which were not consistently at the advantage of one or the other group of participants). Individuals with WS thus exhibit lower numerical and spatial acuity than would be expected on the basis of their verbal developmental level. Predictably, their acuity in the processing of numerosities and spatial magnitudes is rather in keeping with their non-verbal developmental level as participants with WS showed comparable Weber fractions with their non-verbal matched controls.

These results are consistent with previous works which provided evidence of a lower ability to discriminate or compare numerosities in young and older individuals with WS [42], [47]. Our study further indicates that this reduced sensitivity to magnitude variations is not specific to numerical cognition- as it extends to spatial magnitudes- but is not either domain-general-as the processing of temporal information turns out to be preserved compared to verbal matched control children. The presence of differential group effects in each task thus argues against strong theoretical position according to which number, space and time representations would be subserved by a single, fully shared magnitude system [1] (but see [75] for a similar dissociation between number and time processing in dyscalculia). Yet, the presence of similar group differences across the spatial and the numerical comparison tasks is consistent with the hypothesis of a developmental continuity between the processing of spatial and numerical magnitudes in the visual modality.

The lower numerical and spatial acuity indicates that people with WS are not able to represent numerosities and spatial magnitudes with the same precision as their verbal matched TD peers, making them less able to detect the finest variations while comparing numerosities or lengths. The lower resolution in spatial magnitude representation could definitely account for a part of their difficulties when they have to solve more complex visuo-spatial task requiring the mastery of proportions for example. Furthermore, some authors speculated that a lower sensitivity to spatio-temporal variations would have repercussions on numerosity processing [2], [3], [13]. Although the way in which spatial processing actually influences the formation of numerosity representations in the visual modality remains unspecified so far, processing the numerosity of visual arrays must somehow requires operating on the visuo-spatial properties of the collection. One might speculate that the representation of visual numerosity would be derived from a conjunction of visuo-spatial features such as, for example, the ratio between the cumulative area and the average distance inter-stimuli. Supporting this view, Gebuis et al. [76] used event-related potentials to examine the time course of perceptual and numerical magnitude information while processing arrays of dots. Their results clearly demonstrated that perceptual and numerical magnitude processing interacted at the level of stimulus evaluation (affecting the latency of the P3 component), well before the start of selective motor preparation (Response-locked lateralised readiness potential not affected). Accordingly, a lower resolution in spatial magnitude representation should naturally disrupt the development of numerical magnitude representation and impede its refinement with age.

It could be argued that the divergent pattern of group difference across tasks would be an artifact of the working memory differences between groups. Working memory assessment indeed demonstrated that individuals with WS had lower VSSP resources than their verbal matched TD peers but similar phonological loop capacities. The dissociation between these two components of working memory, that is, preserved phonological loop (compared to chronological and developmental age matched children) [14], [30], [77], [78] versus deficient VSSP [79], [80], is recurrently reported in the literature. The group effect in the number and the length comparison tasks would merely reflect the limitation of the VSSP resources in people with WS as the numerical and the spatial tasks both recruit, to some extent, the VSSP. The absence of group difference in the duration comparison task would result of the preservation of the phonological loop capacities. However, this explanation by itself is unlikely to account for the present results. Indeed, in the length comparison task, participants only had to hold a single element in working memory, that is, the length of a single line, and to compare it to the length of the second line. Although WS patients exhibited a reduction of the VSSP capacities, their score in the visuo-spatial span task nevertheless indicated that they are perfectly able to hold more than two elements in their VSSP. The numerical comparison task probably recruits more resources in the VSSP as the position of each individual pieces as to be coded before the numerosity could be extracted [81]. However, there was no correlation between the visuo-spatial span and the Weber fraction within-pair differences neither in the numerical or the spatial comparison tasks.

Another interesting outcome concerns the relationship between the numerical and non-numerical acuity and mathematical performance. Strong correlations have indeed been reported between numerical and non-numerical acuity indexes and the precocious math index that recovers the first acquisitions in mathematical development: the higher the numerical and non-numerical acuity (indicated by a low *w)*, the higher the performance in the give-a-number and the pictorial additive fluency tasks. Surprisingly however, none of the acuity indexes correlated with the arithmetic fluency index after controlling for general cognitive abilities. This contrasted pattern of correlations is partly consistent with the perspective of Walsh [2], [3] and Simon [13] who assumed that number processing development and later mathematical achievement would be rooted in our ability to handle non-numerical magnitudes. Our results indicate that this could be true at least for the first formal numerical acquisitions. Furthermore, our data support the existence of a link between numerical acuity and the acquisition of the first symbolic numerical competences (see [82] for similar results). However, the absence of a correlation between the numerical *w* and the arithmetic fluency index contradicts previous findings showing a significant relationship between non-symbolic numerical acuity and later mathematical achievement [63]–[65], [83] and adds further support to studies which did not find such kind of link [84]–[87]. Regarding the way non-numerical and numerical magnitudes support the acquisition of formal mathematics, it would be useful in the future to consider the multidimensional nature of the mathematics domain. Recently, Lourenco and her colleagues [66] observed that adults’ precision in comparing numerical magnitudes uniquely predicted advanced arithmetic scores in a standardized battery, while their performance in a non-numerical area comparison task was a unique predictor of geometry achievement. However, neither the precision in the numerical nor in the area comparison tasks was related to elementary arithmetic fluency, word problem solving or knowledge of math-related concepts, suggesting that numerical nor non-numerical magnitude might have differential contribution to formally taught mathematics.

As a final point, the present results have at least two implications for studies in the future. First of all, they stressed the importance to focus on basic, low-level spatial dimensions to progress in the understanding of higher level visuo-spatial dysfunction. People with WS’s difficulty to process spatial magnitudes could reasonably be supposed to play a significant role in the global visuo-spatial dysfunction reported in more complex tasks. Indeed, a good grasp of visuo-spatial relationships probably requires having a good estimation of spatial dimensions as well. Actually, there is no other way to appreciate how people with WS process and structure visuo-spatial inputs than to decompose and explore the more basic visuo-spatial processing subcomponents. A systematic examination of low-level visuo-spatial processing in neurodevelopmental syndromes is also a unique opportunity to enrich our knowledge of visuo-spatial cognition and to understand the developmental trajectory that leads to a complex pattern of visuo-spatial dysfunction at the end-state.

Secondly, we can hypothesize that the difficulty of patients with WS to process number magnitude would result from a primitive dysfunction of visuo-spatial magnitude processing. In the case of WS, the very basic difficulty might not be to process number magnitude per se, but to process numerosities presented in space. For now, our results stress the necessity to clarify the origin of their difficulty to process visuo-spatial magnitudes and to determine whether the nature of this deficit is spatial or visual. Under the assumption of a general disability in processing visual magnitudes, participants with WS should also experience difficulty in processing visual duration with no spatial processing requirement such as, for example, the presentation duration of a visual stimulus. Likewise, contrasting non symbolic numerical processing with and without spatial processing requirement within the visual modality would help to determine how far their difficulty to process visual numerosities is grounded in their spatial processing disorder. Observing how they handle numerosities in other sensorial modality could be another way to assess the specificity of their difficulty with number magnitude processing. Of course, these considerations remain speculative and would need to be supported with additional data bringing early and longitudinal evidence of a developmental link between non numerical and numerical acuity development. But it could be suspected that precocious difficulties to process non-numerical magnitudes could prevent children to start their numerical development on solid foundations, making them more at risk to develop mathematical learning difficulties. Further research is necessary to track longitudinally how such basic deficits in processing spatial and/or temporal non numerical magnitudes would impact subsequent numerical development.

In conclusion, our findings stimulate further investigation into the developmental nature of the relationships between numerical and non-numerical magnitudes. They indicate that the magnitude processing deficit in WS is not specific to the numerical domain and extends to spatial magnitudes as well. Whether there is a developmental causality between the reduced acuity in both the spatial and the numerical magnitude processing in the visual modality remains to be established. The longitudinal study of neurodevelopmental disorders at risk to associate numerical and non-numerical magnitude processing deficits (i.e. Williams, velocardiofacial or Turner syndromes) could shed light on the developmental link between these deficits and could provide insight into their trajectory in the course of the development.

## Supporting Information

Supplement S1 **Weber fraction estimation method.**
(DOCX)
