# Supplementary material for: Magnitude Representations in Williams Syndrome: Differential Acuity in Time, Space and Number Processing
Source: PLoS One. 2013 Aug 28;8(8):e72621. doi: 10.1371/journal.pone.0072621 (PMC3755976; doi:10.1371/journal.pone.0072621)
Supplement: Supplement S1 — Weber fraction estimation method. (DOCX) [file pone.0072621.s001.docx]

**Weber fraction.** As predicted, participants’ performance varied as a function of the stimulus ratio (see Figure 2). In order to assess the precision of the underlying magnitude representations, the Weber fraction (*w*) was estimated individually from participants’ correct response distribution in each task. There are several ways to define a Weber ratio and the approach taken here is the one described by [Pica et al. (2004](#_ENREF_60)) and [Halberda and Feigenson (2008](#_ENREF_24)). These authors consider a Gaussian distribution for the internal representation of the two magnitudes to be compared (*m*_1_ and *m*_2)_ and they derive an expression of the error probability which can be converted to a probability of giving a correct response. The resulting formula for the probability of error is

$$\frac{1}{2}erfc\left( \frac{m2-m1}{\sqrt{2}w\sqrt{m_{1}^{2}+m_{2}^{2}}} \right),$$

corresponding to the probability of success (a)

$$\Phi\left( \frac{\frac{m_{2}}{m_{1}}-1}{w\sqrt{1+\left( \frac{m_{2}}{m_{1}} \right)^{2}}} \right).$$

Here, *m*_1_, *m*_2_ and $\Phi$ represent the smallest magnitude, the largest magnitude and the cumulative standard normal distribution function respectively; the parameter *w* is the Weber fraction. The argument of this expression becomes 1 when

$$w=\frac{\frac{m_{2}}{m_{1}}-1}{\sqrt{1+\left( \frac{m_{2}}{m_{1}} \right)^{2}}}$$

The corresponding value of the cumulative standard normal distribution is approximately equal to 84.1%. This allows a graphical interpretation of the Weber fraction as the value of

$$\rho=\frac{\frac{m_{2}}{m_{1}}-1}{\sqrt{1+\left( \frac{m_{2}}{m_{1}} \right)^{2}}}$$

at which the success rate is $\Phi\left( 1 \right)\approx84.14\%.$ This can be used to determine the Weber fraction empirically if the corresponding experiments included magnitude ratios ${m_{2}/m}_{1}$ whose corresponding success rates surround and are near to 84.14%. The method used here for determining the Weber fraction is the method of the maximum likelihood. At any value $\rho_{i}$ corresponding to a magnitude ratio, the probability of observing $k\_i$ successes on $n_{i}$ test items is given by a binomial distribution

$$\left( \begin{matrix} n_{i} \\ k_{i} \end{matrix} \right)p_{i}\left( w \right)^{k_{i}} \left( 1-p_{i}\left( w \right) \right)^{n_{i}-k_{i}}$$

where $p_{i\left( w \right)}=\Phi\left( \rho_{i}/w \right)$. This yields the combined likelihood over all ratios

$$Lik\left( w;data \right)=\prod\left( \begin{matrix} n_{i} \\ k_{i} \end{matrix} \right)p_{i}\left( w \right)^{k_{i}} \left( 1-p_{i}\left( w \right) \right)^{n_{i}-k_{i}}.$$

This likelihood can now be maximized (more conveniently on the log scale) to determine the maximum likelihood estimate of w. It is clear that such an estimate is ill-defined when the rate of correct responses for all magnitude ratios is much lower than 84.13%. In such cases the maximum likelihood estimate of *w* may turn out unbounded or unreasonably high. This can be avoided by adding a magnitude ratio at which all subjects obtain nearly perfect results. In our study, this was implemented as a pre-test. Here the magnitude ratio was 3 and obtaining at least 5 out of 6 correct responses was the entry criterion for the main trial. The probability of obtaining at least 5 out of 6 correct responses at a magnitude ratio of 3 is

$$6\cdot p_{i}\left( w \right)^{5}\cdot\left( 1-p_{i}\left( w \right) \right)+p_{i}\left( w \right)^{6}=p_{i}\left( w \right)^{5} (6-5{\cdot p}_{i}\left( w \right)^{5})$$

which can be added to the likelihood and serves to stabilize the calculation. This method permits therefore to estimate individual Weber fractions for all individuals who passed the pre-test. Note that the pre-test can add a bias to the estimation when the true Weber fraction is very large. Here the probability to pass the pre-test is low and therefore subjects who have very low abilities to discriminate are excluded. In order to assess this bias, we carried out a simulation study. This simulation study showed no or almost no bias for Weber fractions between 0.05 and 0.5. For Weber fractions above 0.6 (corresponding to a magnitude ratio of almost 3/1), bias increases and becomes non-negligible above 0.7. This is that the procedure under-estimates the Weber fraction for very severely impaired subjects. In the context of the present study, such subjects were not part of the target population. The abovementioned estimation bias is therefore not relevant in our situation.
